# Functional Interviewing Was Associated With Improved Agreement Among Expert Psychiatrists in Estimating Claimant Work Capacity: A Secondary Data Analysis of Real-Life Work Disability Evaluations

**DOI:** 10.3389/fpsyt.2020.00621

**Published:** 2020-07-03

**Authors:** David Y. von Allmen, Sarah Kedzia, Raphael Dettwiler, Nicole Vogel, Regina Kunz, Wout E. L. de Boer

**Affiliations:** Evidence-based Insurance Medicine, Department of Clinical Research, University of Basel and University Hospital of Basel, Basel, Switzerland

**Keywords:** disability evaluation, independent medical evaluation, work capacity evaluation, mental disorders, International Classification of Functioning, Disability and Health, evidence-based medicine, evaluation studies, work participation

## Abstract

**Objective:**

Functional evaluations establish functional and work (in-)capacities in the context of disability assessments and are increasingly recommended as a modern technique for work disability assessments. The RELY (Reliable disability EvaLuation in psychiatrY)-studies introduced semi-structured functional interviews in real-life assessments of claimants with mental disorders for evaluating their self-perceived health-related limitations and for investigating the reproducibility of work capacity (WC) estimates. Functional interviews elicit claimants’ self-perceptions about their work-related limitations and capacities in the labour market. This secondary data analysis explored the coverage of work-related key topics in these interviews and investigated whether interviews with high coverage (versus low coverage) of work-related topics resulted in better reproducibility of WC estimates among experts.

**Methods:**

Thirty video-taped RELY-assessments underwent a content analysis along a predefined framework for functional interviewing, including the claimant’s self-perceived work limitations and work-related health complaints as centrepieces of functional interviewing. Following transcription, interviews were segmented into coding units. Coding units were allocated to the five steps with 19 key topics of the framework. Enquiry into key topics was ascertained by summing the functional coding units per key topic. Median split grouped the interviews into high and low coverage of functional topics and compared them for inter-rater reliability (intraclass correlation coefficient, ICC) and inter-rater agreement (standard error of measurement, SEM).

**Results:**

Interviews were broken down in 40,010 coding units, 31% of which addressed functional topics. Enquiries in self-perceived work limitations and work-related health complaints were sparse (coding units median_psychiatrist_ between 0 and 1.5, median_patients_ between 0 and 9.5). High coverage interviews enquired on more functional topics (68% vs. 42%, chi^2^(1, N = 38) = 5.32, p = 0.021) and in more depth (36% vs. 16% of functional coding units, chi^2^(1, N = 1,314) = 141.15, p < 0.001). Interviews with higher functional coverage reached significantly higher inter-rater agreement in WC ratings among experts (mean difference in SEM, low–high coverage, 7.5% WC, 95% CI 0.2 to 15.1%WC). Inter-rater reliability was low in both groups (ICC, 0.38 versus 0.40).

**Conclusions:**

Content analysis showed little enquiry by experts on claimants’ self-perceived activity limitations and work-related capacity. The association between interviews with higher functional coverage and better expert agreement on the claimants’ remaining WC requires confirmation in prospective studies.

## Highlights

**What Is Known?**

- Work disability evaluations are frequently criticised for their lack of transparency how experts derive their judgement.
- Modern thinking of work disability suggests functional evaluations as the way to move forward. There, claimants are assessed for work-related capacities and activity limitations.
- A recent study showed low reproducibility (i.e., inter-rater reliability^1^ and inter-rater agreement^2^) of work capacity judgements among psychiatric experts despite training in functional interviewing.

**What Does This Study Contribute?**

- In RELY 1, experts barely explored claimants’ self-perceived activity limitations and work-related capacities, both central elements of functional interviewing.
- Interviews that addressed more work-related functional topics were associated with higher expert agreement on the claimants’ remaining work capacity.
- The findings encourage to further study the impact of functional interviewing on expert agreement following refinement and timely provision of training.

## Introduction

Having recognised that impairment-based assessments are poor proxies for an individual’s capacity to work (1, 2), modern thinking of work disability assessment in insurance medicine has shifted towards functional evaluations (3, 4) as a way to move forward. The term ‘functional evaluation’ indicates a change in focus from the biomedical approach which considers work disability as a characteristic of an individual with impaired health to the biopsychosocial approach of the International Classification of Functioning, Disability and Health (ICF) (5). There, work disability is thought as the result of the interaction between an individual’s impaired health with work requirements and other factors. In his recent comparison of international developments on work disability evaluations, Baumberg Geiger identified three models of directly assessing work capacity (WC) that were implemented to varying degrees in national practice (6): a) “demonstrated assessments” that use claimants’ experience in the labour market; b) “structured assessments” that match functional requirements to workplace demands; and c) “expert assessments” that integrate the judgments of skilled professionals. All three models tend to integrate specific techniques to assess the claimants’ work-related capacities and their activity limitations in a work environment.

**Figure 1:**
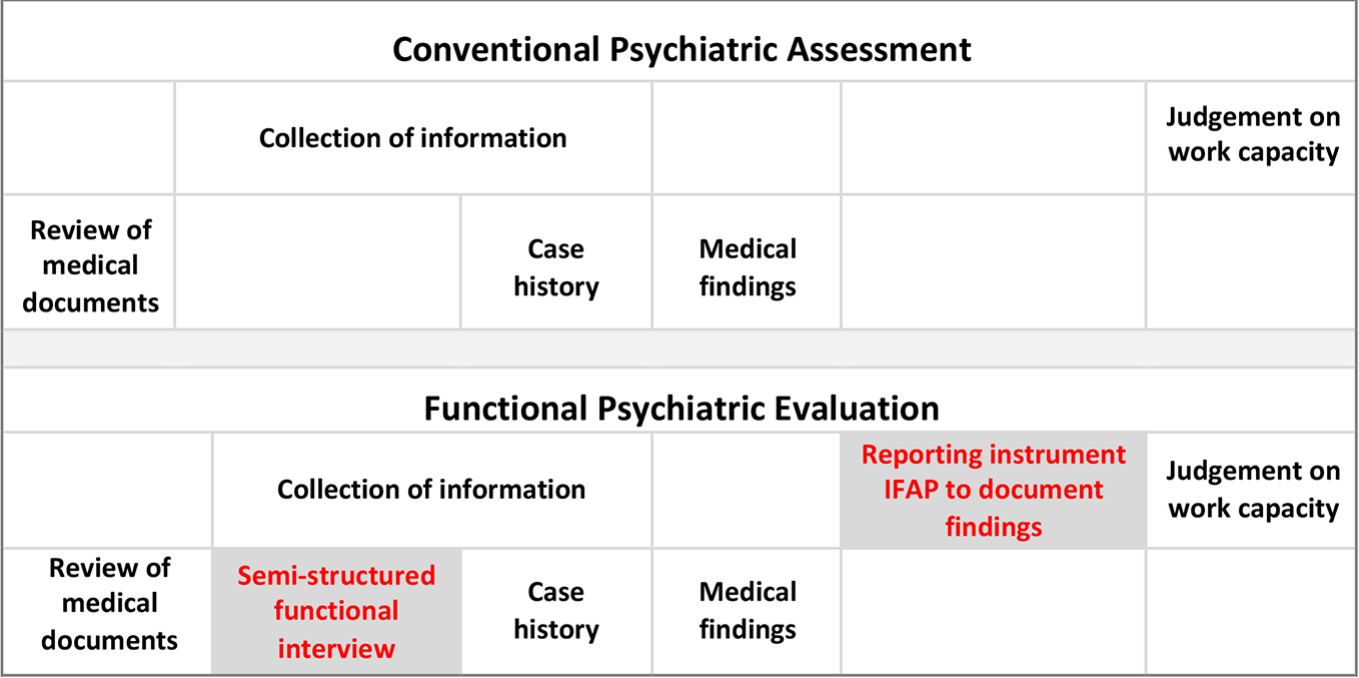
Functional psychiatric evaluation complements conventional psychiatric assessment. Functional psychiatric evaluation consists of a semi-structured functional interview to elicit the patients’ functional limitations and remaining capacities and the Instrument for Functional Assessment in Psychiatry (IFAP) to document them with reference to common work-related activities (17).

Switzerland, one of the countries that promotes such a shift (1, 7–11) is suggesting the ICF as a reference framework to establish and communicate functional and work (in-) capacity in the context of disability assessments (8, 9, 12, 13). However, its implementation into practice is still at the beginning (4, 7, 14). Our novel concept of functional evaluation complements current psychiatric assessment practice by components from all three models (4, 15, 16): First, a semi-structured functional interview which elicits claimants’ self-perceptions about their work-related limitations and capacities in the labour market, second, an instrument (‘Instrument for Functional Assessment in Psychiatry’, IFAP) to document these limitations and capacities in a structured way with reference to workplace demands, third, performed by experts who had undergone skills training in performing functional evaluations (**Figure 1**).

Based on Dutch examples (16), we developed a five step protocol for functional interviewing (15, 18): Orientation about the upcoming assessment; the patients’ last job and previous work activities; the patients’ self-perceived work limitations; their work-related health complaints; summary by the psychiatrists about their understanding of the patients’ self-report (**Table 1**). Scheduled very early in the course of the psychiatric assessment, the patients’ self-perception serves as a reference for the psychiatrists to validate during the remaining assessment. After the functional interview, psychiatrists continue their assessment according to their personal routine.

**Table 1:** Purpose and content of functional interviewing: The five steps and the category system with 19 key topics.

Purpose and content	Key topics and residual categories	Descriptions and examples
**Step 1 Orientation:** Orientation about the assessment, including a short introduction, general regulations and the interview agenda	1. Opening	Psychiatrist introduces him-/herself.
	2. RELY study	Psychiatrist provides patient with information about RELY 1.
	3. General regulations	Psychiatrist provides patient with information about regulations within disability assessment.
	4. Interview agenda	Psychiatrist provides patient with an outline about the topics to clarify in the forthcoming interview.
	Residual category (orientation)	Information addressed within orientation, but not associated with the preceding four topics.
**Step 2** **Last job and previous activities:** Enquiry about the last job and specific activities in the job, to establish a basis of information for the assessment of work capacity	5. Job title/education	Past job titles and education completed (e.g., construction worker).
	6. Employer	Past employers (e.g., company name, location).
	7. Employment duration	Time spans worked in past employments.
	Residual category (job)	Information associated with past jobs, but not assignable to the preceding three topics (e.g., experience of working atmosphere, mobbing).
	8. Previous activities	Specific activities performed in the last job (e.g., cleaning scarf boards)
	9. Feeling towards activities	Feelings while performing the activities (e.g., joy or boredom)
	Residual category (activities)	Information associated with previous activities, but not assignable to the preceding two topics (e.g., priorities among activities, task difficulty)
**Step 3** **Self-perceived work limitations**: Enquiry about the possibility to work in the last job and in a suitable alternative work, and conditions for a successful performance	10. Previous activities	Possibility to successfully performing previous activities.
	11. Suitable alternative activities	Possibility to successfully performing suitable alternative activities.
	12. Conditions for successful performance	Conditions necessary for successful performance of activities (e.g., short breaks).
**Step 4 Work-related health complaints and symptoms**: Enquiry about health complaints affecting work performance and to substantiate the claimed work limitations	13. Specific work-related health complaints	Experienced symptoms (type, localisation, severity) during work (e.g., strong backache).
	14. Exacerbating and attenuating circumstances	Circumstances that improve/worsen symptoms (e.g., weight to lift, work duration)
	15. Emotional-cognitive coping	Reaction to the experience of symptoms (e.g., interruption, resistance, avoidance, prevention).
	Residual category (work-related health complaints)	Information associated with work-related health complaints, but not assignable to the preceding three topics
**Step 5** **Summary**: Summary and clarification of circumstances that prevent the claimant from working	16. Last job	…
	17. Previous activities	…
	18. Self-perceived work limitations	…
	19. Work-related health complaints	…

During a specially designed training programme in functional evaluation, psychiatrists affiliated with four assessment centres engaged in small-group training with three sessions of three hours each, over a period of three months. The training used lectures, an instruction text, role play, plenary and small group discussion, and homework to practice the new skills (19).

The RELY (Reliable disability EvaLuation in psychiatrY)-studies (17, 19) were designed to establish the reproducibility [i.e., inter-rater reliability and inter-rater agreement (20)] of the functional evaluation^3^. Thirty real-life claimants recruited from the national disability insurance underwent real-life work disability assessments by 12 expert psychiatrists trained in functional evaluation who determined the claimants’ WC. The assessments were videotaped and used by other psychiatrists (‘rating psychiatrists’, three per patient) likewise trained in functional evaluation to independently determine the WC of this patient, resulting in four WC ratings per patient. RELY 1 established that functional evaluation demonstrated low inter-rater reliability to discriminate between claimants with low, moderate, and high WC (intraclass correlation coefficient, ICC, 0.43; 95% CI 0.22 to 0.60) and low inter-rater agreement among experts about claimants’ remaining WC (standard error of measurement, SEM, for WC was 24.6%, 95% CI 20.9 to 28.4) (17, 21).

Since two major administrative changes by the Swiss government had interfered with patient recruitment causing a training-to-interview delay of about a year (median = 342 days), the study results were ambiguous to interpret: Had we erred on the concept of functional interviewing or had the training been insufficient? We therefore performed a content analysis of the RELY assessments to examine to what extent expert psychiatrists had applied functional interviewing and in what depth psychiatrists and patients had delved into functional key topics and compared the impact of interviews with low coverage versus those with high coverage of functional topics on reproducibility (i.e., inter-rater reliability and inter-rater agreement) of WC ratings.

## Methods

### Study Design and Participants

In this cross-sectional study, we performed a content analysis of real-life work disability assessments to examine expert psychiatrists’ adherence to the functional interviewing protocol and investigated whether interviews with high coverage of work-related functional topics (versus low coverage) resulted in better reproducibility of remaining WC among experts. The data were derived from a previous multi-centre study [RELY1-study (17, 19)] that determined the reproducibility and transparency of psychiatric disability assessments in social insurance.

Psychiatrists were eligible to participate in RELY 1 if they worked for one of the following assessment centres: Department of Insurance Medicine, University Hospital Basel, Centre for Medical Assessments Basel, MEDAS Central Switzerland, MEDAS Interlaken, or for the Suva Clearinghouse. Patients who had applied for disability benefits at the Zurich office of the Invalidity Insurance were eligible to participate if they were German-speaking and scheduled for a polydisciplinary disability assessment, including a psychiatric assessment. The recruitment procedure was published in detail elsewhere (17, 19).

### The Content Analysis

#### Category System

We defined 19 functional interview topics (referred to as ‘key topics’; information about last job and previous activities, self-perceived work limitations and work-related health complaints), plus four residual categories (**Table 1**). Key topics capture information considered to be essential for the assessment of WC (15, 16). We extended the category system by 15 medical/general issues frequently addressed in disability assessments which cover medical complaints not specifically linked to work [i.e., underlying cause, interventions and rehabilitation, health-related, psychosocial and biographical information, plus two residual categories (16); **Supplementary Table 1**]. We followed the principle to keep both category systems mutually exclusive. In the context of this study, we only report summary findings. The methodology of functional interviewing schedules the claimants’ self-perceived work limitations at the beginning of the work disability assessment to provide the psychiatrists with a reference for their assessments. To prevent missing functional interviews that were placed early but not at the very beginning of the assessment, we extended the content analysis to the first hour of the interview.

#### Coding Procedures

We recorded all interviews on video. After verbatim transcription (RD) (22), we segmented the transcripts into separate coding units after each change of speaker, punctuation mark (full stop, question mark, comma), grammatical conjunction (‘and’, ‘or’) and restarts (cutting off or rephrasing utterance). That way, the segmentation process was kept separate from the coding process (23). A coding unit was defined as the ‘minimal textual component’ assigned to one of the 19 key topics (functional coding unit) or to one of the 15 medical and general issues (medical/general coding units). In case our systematic segmentation produced coding units that were meaningless on their own, we coded these units according to the context. For instance, when a single unit carried no information for assignment, e.g. an affirmative “Yes”, we coded according to the preceding question. Furthermore, we developed coding rules to clarify how to deal with the overlap in specific situations (e.g. ‘work-related health complaints’ versus ‘health complaints not related to work’). We used three transcribed interviews to pilot the category systems and the coding rules. We coded in pairs (DA, RD, SK), all of whom had been involved in the development of the category system and calculated the intercoder reliability using Krippendorff’s Alpha (α) (24, 25). An α of 0.80 is often considered as the norm for good reliability, with a minimum of α = 0.67 for tentative conclusions (26). Having calculated the intercoder reliability, we solved coding discrepancies in a consensus group consisting of one senior expert researcher (WB) and two coders (DA, SK).

#### Data Analysis

A key topic was considered as being ‘covered’ by either psychiatrist or patient if at least one functional coding unit had been allocated to that key topic. We calculated coverage of key topics, i.e., number of key topics addressed by at least one functional coding unit, identified key topics that were covered poorly or not at all, and determined in what depth psychiatrists enquired into WC by calculating the number of functional coding units per key topic (median m_coding.unit_, interquartile range IQR), for psychiatrists and patients. To assure readability, we limited reporting of the IQR to the tables.

### Comparing Interviews With High Versus Low Coverage of Functional Key Topics

#### Topics Covered and Omitted: A Descriptive Analysis

In a secondary data analysis, we compared interviews with high versus low coverage of key topics in analogy to the main analysis: a) number of key topics covered, b) kind of key topics poorly or not at all covered, and c) number of functional coding units per topic to determine the depth of topic coverage, resp. medical/general coding units. We defined interviews with high and low coverage of key topics by assigning one point for each key topic (**Table 1**) addressed by psychiatrist or patient at least once (sum score between 0 and 38), rank-ordered them and grouped them by median split. We report the functional coding units for psychiatrists and patients separately.

To test whether our procedure to determine ‘coverage’ reflected the amount of information obtained on WC (i.e., number of coding units per key topic), we calculated a simple linear regression to predict the number of functional coding units assigned to key topics per interview (dependent variable) based on the number of key topics covered (independent variable). We interpreted the linear relationship coefficient (R) as small (0.10 to 0.29), medium (0.30 to 0.49), and large (≥0.50) (27).

#### Reproducibility of Work Capacity Ratings

Data collection of expert judgement of patients’ WC was published in detail elsewhere (17, 19). To determine reproducibility, variance components (psychiatrists, patients, residuals) underlying the ICC and SEM were estimated using a linear mixed-effects model. The model used WC as response variable and crossed random intercepts for patients and psychiatrists. An intercept was fitted as the only fixed effect. Each pair of datasets (high coverage interviews and low coverage interviews) was compared by fitting the linear mixed-effects models and by calculating the differences in ICC and SEM (low coverage minus high coverage interviews). We used model-based bootstrapping for both, estimation of 95% CI of the ICC and group comparisons. The procedures were repeated 9,999 times. ICC is reported as a ratio between 0 and 1, SEM in the natural units % WC. Lower values of SEM indicate higher agreement. We used Student t-test and chi^2^-test to compare continuous and categorical variables, respectively.

In the *Results* section, we restrict our report to the primary outcome ‘work capacity for alternative work’ WC_alternative.work_, and the related measure of inter-rater agreement (SEM) and inter-rater reliability (ICC) (17, 19). In **Supplementary Table 2**, we report the results for both outcomes, WC_last.job_ and WC_alternative.work_, in analogy to the main paper (17).

### Telephone Survey on the Psychiatrists’ Perceptions of Functional Interviewing

To put the content analysis into context, we compared its findings on functional interviewing with the psychiatrists’ self-report: Following the RELY 1-evaluations, an independent psychiatrist had conducted a semi-structured telephone survey (7 questions) among the 12 interviewing psychiatrists (19) to elicit their perception on the functional evaluation, including three open questions on the functional interview: ‘Did you use the functional interview in the RELY-study?’, ‘Have you benefitted from the training in functional interviewing?’, ‘Do you keep using it in your practice?’ The survey was audiotaped, the responses transcribed and categorized using an ad-hoc Yes/No-scheme.

## Results

### Psychiatrist and Patient Characteristics

Twelve psychiatrists performed 30 interviews for assessing work disability. Most were middle-aged (41–60 years, 63%) or older (32%) and male (79%). Their professional experience in performing disability evaluations was 13.8 years (mean, SD = 9.2). The patients’ age was 47.2 years (mean, SD = 8.6), 57% were male. The most common diagnoses were mood disorders (26%), followed by neurotic, stress-related and somatoform disorders (19%). Detailed psychiatrist and patient characteristics are listed in **Supplementary Table 3**.

### General Description of the Content Analysis

The first 60 minutes of the 30 assessment interviews contained 40,010 coding units, of which 31% were assigned to functional key topics and 69% to medical/general issues (**Table 2**). Assessments addressed 61% (23/38) of functional key topics (median) with similar coverage by psychiatrists (63%) and patients (58%). Assessments used 26% of coding units for functional key topics and 77% for medical/general coding units. Intercoder reliability α was 0.72 (95% CI 0.71 to 0.73). **Supplementary Table 4** provides exemplary outtakes on functional interviewing from a RELY 1-assessment.

**Table 2:** Time and effort spent on work-related functional key topics during the interview.

Summary of findings	All interviews (N = 30)				High coverage interviews(N = 15)				Low coverage interviews(N = 15)			
Key topics per interview covered by psychiatrists and patients (n = 38)	61% IQR: 41–71%				68% IQR: 63–74%				42% IQR: 29–55%			
Coding units per interview assigned to functional key topics	26% (346/1,314) IQR: 16–41%				36% (475/1,309) IQR: 25–48%				16% (210/1,318) IQR: 11–31%			
Coding units per interview assigned to medical and general issues	77% (1,011/1,314) IQR: 52–92%				59% (772/1,309) IQR: 47–87%				83% (1,100/1,318) IQR: 60–99%			
					**Functional coding units**							
**Interviewing steps and key topics**	Psychiatrists		Patients		Psychiatrists		Patients		Psychiatrists		Patients	
	Median	IQR	Median	IQR	Median	IQR	Median	IQR	Median	IQR	Median	IQR
**Step 1: Orientation**												
Opening	0	0–0	0	0–0	0	0–0	0	0–0	0	0–0	0	0–0
RELY study	3.5	0–7	0	0–3	4	0–7	2	0–4	3	0–6	0	0–1
General regulations	4	1–12	1	0–5	3	1–13	1	0–2	5	0–10	2	0–8
Evaluation agenda	9.5	1–15	0	0–1	10	1–17	1	0–2	9	3–14	0	0–1
**Step 2: Last job and previous activities**												
Job title and education	2	0–5	3	1–9	3	1–5	3	1–10	2	0–6	3	1–8
Employer	1	0–4	3.5	1–5	1	0–3	4	1–6	1	1–4	2	1–4
Employment duration	3.5	1–7	5	3–9	3	2–5	5	4–8	4	1–10	5	2–11
Previous activities	6.5	2–15	18.5	10–27	8	2–15	20	11–30	4	2–13	16	10–24
Feelings towards activities	0	0–1	2.5	0–6	0	0–2	5	1–8	0	0–1	1	0–4
**Step 3: Self-perceived work limitations**												
Previous activities	0	0–4	1	0–5	3	0–8	5	2–9	0	0–1	0	0–0
Suitable alternative activities	0	0–6	0	0–4	4	0–8	3	0–9	0	0–0	0	0–0
Conditions required for successful performance	0	0–6	1	0–12	4	1–12	9	4–22	0	0–0	0	0–0
**Step 4: Work-related health complaints**												
Complaints and symptoms	11.5	0–45	32	4–73	30	11–51	54	31–81	0	0–16	3	0–34
Exacerbating and attenuating circumstances	0	0–3	1	0–10	3	1–12	9	2–24	0	0–0	0	0–0
Emotional-cognitive coping	1.5	0–11	9.5	0–30	5	1–20	28	8–61	0	0–7	0	0–13
**Step 5: Summary**												
Last job	0	0–1	0	0–0	0	0–2	0	0–0	0	0–1	0	0–0
Work activities	0	0–0	0	0–0	0	0–0	0	0–0	0	0–0	0	0–0
Self-perceived work limitations	0	0–0	0	0–0	0	0–0	0	0–0	0	0–0	0	0–0
Work-related health complaints	0	0–5	0	0–0	0	0–17	0	0–1	0	0–0	0	0–0

The transcripts of 30 assessment interviews contained 40,010 coding units, 31% were assigned to the 19 functional key topics and 69% to the 15 medical/general issues. The number of functional coding units used by psychiatrists and patients document the degree of work-related enquiry. Step 3 ‘Self-perceived work limitations’ and step 4 ‘Work-related health complaints’ constitute the centrepiece of functional interviewing. The number of key topics addressed was used to distinguish interviews with high versus low coverage (Median split). For the summary of findings, results are reported as median in % and interquartile range, IQR.

### Enquiry Into Functional Key Topics Delineating Functional Interviewing

#### Step 1: Orientation

Psychiatrists provided a short introduction and orientation on the RELY-studies, general regulations, and the evaluation agenda (m_coding.unit_: 3.5; 4; 9.5 respectively) (**Table 2**).

#### Step 2: Last Job and Previous Activities

Any enquiry of WC requires some background about previous jobs and specifications of the work activities. Psychiatrists conducted a balanced interview with the claimants covering almost all topics: job title, employer details, employment duration, activities, and feelings towards these activities. The medians of coding units ranged between 0 and 6.5 per topic for psychiatrists and between 2.5 and 18.5 for claimants.

#### Step 3 and 4: Self-Perceived Work Limitations and Work-Related Health Complaints

Self-perceived work limitations and work-related health complaints are the centrepiece of functional interviewing where psychiatrists are expected to get to the bottom of what prevents the claimant from working. Self-perceived work limitations have barely been addressed, neither by psychiatrists (m_coding.unit_: 0 for previous activities, suitable alternative activities, and conditions necessary for successful performance) nor by claimants (m_coding.unit_: 1 for previous activities, 0 for suitable alternative activities, and 1 for conditions necessary for successful performance). Enquiry into work-related health complaints was slightly more informative for complaints and symptoms (m_coding.unit_: 11.5 by psychiatrists; 32 by claimants), exacerbating and attenuating circumstances (m_coding.unit_: 0 by psychiatrists; 1 by claimants), and emotional-cognitive coping (m_coding.unit_: 1.5 by psychiatrists; 9.5 by claimants).

#### Step 5: Summary

Once claimants had described their self-perceived work limitations and work-related health complaints, the methodology of functional interviewing expects the psychiatrists to provide a summary of the information for clarification to ensure a common understanding of the claimants’ self-perception. This was quasi non-existent (m_coding.unit_: 0 for last job, work activities, self-perceived work limitations, and work-related health complaints).

#### Interviews With Low Coverage Versus High Coverage of Functional Topics

Patients undergoing low versus high coverage interviews did not differ with regards to age (47.9 vs. 46.5 years, t(28) = 0.46, p = 0.65) and gender (male, 53% vs. 60%, chi^2^(1, N = 30) = 0.17, p = 0.71) (**Table 2**).

#### Functional Topics Covered and Omitted: A Descriptive Analysis

Assessments with low coverage addressed 42% (16/38) of functional key topics, with an in-depth enquiry of 16% functional coding units. In contrast, those with high coverage addressed 68% (26/38) of functional key topics (i.e., increase by factor 1.62, chi^2^(1, N = 38) = 5.32, p = 0.021) with an in-depth enquiry of 36% functional coding units (i.e., increase by factor 2.25, chi^2^(1, N = 1,314) = 141.15, p < 0.001. The strong relationship between the number of key topics and the number of functional coding units assigned to these key topics (R = 0.76, p < 0.001 and the disproportionate increase in functional coding units observed with increasing number of key topics indicates that psychiatrists and patients who covered more key topics in the functional interview explored each topic in more details (**Supplementary Figure 5**).

Interviews with low and high coverage did not differ much in length regarding orientation and enquiry of last job and previous activities. They differed, however, with regards to self-perceived work limitations where interviews with low coverage of key topics failed to enquire into previous activities (m_coding.unit_ low vs. high: 0 vs. 3 by psychiatrists; 0 vs. 5 by patients), suitable alternative activities (0 vs. 4 by psychiatrists; 0 vs. 3 by patients), and work-related health complaints (0 vs. 30 by psychiatrists; 3 vs. 54 by patients, **Table 2**). The psychiatrists’ summary on last job, work activities, self-perceived work limitations, and work-related health complaints was quasi non-existing in both groups (m_coding.unit_: 0 by psychiatrists and patients alike).

#### Impact on Reproducibility of Work Capacity Ratings and Level of Work Capacity

Experts who performed interviews with higher coverage of the functional key topics reached significantly higher inter-rater agreement with their colleagues on WC ratings (SEM 20.6% WC vs. 28.1% WC, lower SEM-values indicating better agreement; mean difference of SEM: 7.5% WC, 95% CI 0.2 to 15.1% WC) and attributed claimants a significantly higher level of WC (WC_alternative.work_ 63.0% vs. 46.1%, mean difference: 16.9% WC, 95% CI 6.1 to 28.9) than their colleagues whose interviews covered functional key topics inadequately (**Table 3**). The inter-rater reliability parameter ICC was low in both groups (low vs. high coverage: 0.40 vs. 0.38, 0.02 ICC, estimate difference, 95% CI −0.35 to 0.41), indicating that high coverage interviews were not better suited than low coverage interviews to distinguish claimants with high WC from those with low WC.

**Table 3:** Impact of low coverage versus high coverage interviews on estimated work capacity (WC), inter-rater agreement (standard error of measurement, SEM) and inter-rater reliability (intraclass correlation coefficient, ICC).

	Low coverage interviews(95% CI)	High coverage interviews(95% CI)	Difference (Low coverage–high coverage interviews) (95% CI)
			
**Work capacity estimates,** expressed in % WC	46.1% WC (31.0 to 61.2)	63.0% WC (52.6 to 73.4)	−16.9% WC (−6.1 to −28.9)
			
**Inter-rater agreement** as SEM, expressed in % WC	28.1% WC (22.1 to 34.2)	20.6% WC (16.3 to 25.0)	7.5% WC (0.2 to 15.1)
			
**Inter-rater reliability** as ICC expressed as ratio from 0 to 1	0.40 (0.10 to 0.63)	0.38 (0.07 to 0.62)	0.02 (−0.35 to 0.41)

### Telephone Survey Among Psychiatrists

Ten of the 12 psychiatrists who had conducted 27 (90%) of the work disability evaluations confirmed the use of the functional interview protocol in RELY 1, nine psychiatrists declared having benefitted from the training in functional interviewing and reported continued use in their practice.

## Discussion

### Key Findings

This is the first content analysis on real-life work disability assessments in 30 claimants with mental disorders illustrating current practice of functional interviewing on work-related issues. Interviews covered 61% of predefined functional topics during the first hour of the assessment. The majority of experts failed to elicit claimants’ self-perceived work limitations and work-related health complaints, both centrepieces of functional interviewing. Experts who rated interviews with high coverage of functional topics achieved significantly better inter-rater agreement on the claimants’ WC and attributed significantly higher level of remaining WC than experts with low coverage interviews. Inter-rater reliability was poor in both groups.

### Strengths and Limitations

We conducted a content analysis of real-life work disability assessments in a heterogeneous group of patients with mental disorders performed by a broad spectrum of psychiatric experts (17). This approach ascertains that insights from the analysis are applicable to real-life. Our model for functional interviewing (15, 16) had explicit instructions to start by clarifying patients’ self-perceived inability to work which should be further examined in the course of the assessment. Extending the content analysis to the first hour of the interview ensured that we did not miss functional interviews that were placed early but not at the very beginning of the assessment. Both features—generalisability to the real world and extensive recording of the interview—strengthen the appropriateness of the comparison ‘high versus low functional coverage’ and the credibility of the finding that agreement among experts improved with high functional coverage in the interviews.

Low coverage of functional key topics was associated with lower levels of estimated WC. One explanation could be that an interviewing expert’s prior judgments of the claimant’s WC from medical files and other documents shaped the enquiry on work issues. As a consequence, the effort to probe on work-related issues might have been less in claimants with more severe impairments than in those perceived as less impaired. Higher ambiguity in prior knowledge about a patient’s disability to work might have fostered the interviewing expert’s endeavour for intensive enquiry of work issues. This is all the more significant as functional interviewing has the potential to improve agreement in more vague data situations. However, it is noteworthy that other studies observed that functional interviewing detected more activity limitations (28, 29).

The lack of adherence to functional interviewing allows two interpretations: The one-year time gap between training and implementation in the study had caused a substantial decline in the previously acquired skills of functional interviewing. Alternatively, the training had been insufficient in the first place. Both interpretations would support our scepticism that the content analysis rather documents current practice of psychiatric work disability assessments than functional interviewing (17).

By using a single functional coding unit to define ‘coverage of a functional topic’, our threshold to call a key topic ‘covered’ was low. It would be overoptimistic to assume that a single functional coding unit would comprehensively encompass the content of a key topic. The disproportionate increase in functional coding units, however, along an increase in functional key topics assures that ‘number of functional topics covered’ can be considered as surrogate for a comprehensive assessment in this study.

### Functional Interviewing

Research on sickness certification reports about general practitioners’ reluctance to shift from the description of symptoms and underlying mechanisms towards a functional and work-related perspective (30, 31). Lack of training, lack of guidance about work-related health and lack of knowledge about work requirements in today’s working world were named as main barriers. Training experts along structured protocols (32, 33) increased their knowledge and skills to obtain functional information for well-founded judgements on functional ability, and improved self-efficacy in performing functional evaluations (32, 34).

Functional interviewing has proven merits: a recent cluster-randomised trial on injured workers claiming work disability benefits compared a formal 2-day functional capacity evaluation with semi-structured functional interviews of 1.5 to 3 h duration. Assessment based on functional interviews versus those based on functional capacity evaluation showed similar results on all main outcomes, i.e. return-to-work recommendations by the clinicians, effective return-to-work and sustained work level at 1, 3 and 6 months post-assessment, and compensation outcomes for the insurers (35, 36).

The content analysis revealed important gaps about the collection of work-related functional information in RELY 1, which is crucial for well-founded WC assessments and a prerequisite for applying any ICF-measures and -instruments (37). The gaps help to explain the findings in RELY 1 with regards to the low level of agreement among experts when judging the degree of work (in-)capacity in the same patient. The content analysis ruled out the notion that functional interviewing had resulted in poor reproducibility despite successful training. Subjecting the RELY 2-study with its more intensive training programme and timely implementation in the study to a similar content analysis might help to define training needs with regards to content, duration and training techniques.

### Self-Perception Versus Objective Findings

In the RELY 1-survey about endorsement of functional interviewing and its implementation during the study and otherwise (17), the vast majority of psychiatrists (83%) had confirmed its implementation in the study and reported continued use in usual practice. Based on these assertions, we would have expected a larger coverage of functional key topics in the content analysis and better adherence with the functional interview. However, our study highlights the discrepancy between self-perception versus objective findings and documents the need to monitor skills and appropriate implementation of functional evaluation training in routine practice.

### Implications for Policy, Practice, and Research

The overarching goal of work disability assessments is appropriate allocation of societal resources to those who lost their capacity to earn their own living and prevention of inappropriate allocation to those with remaining WC. Our content analysis indicated that the RELY 1-interviews rather reflect current practice than functional evaluation as planned. Despite these shortcomings in practice, it was possible to demonstrate that WC assessments with higher coverage of functional topics achieved substantial improvement in agreement when experts determined the remaining WC of claimants. While such findings seem plausible, it would be important to confirm these findings in a second independent sample, e.g., the interviews performed in the RELY 2-study (17). Furthermore, despite substantial improvement in agreement, the observed level is still far below the expectations of more than 700 Swiss stakeholders who considered a level of 9.0% WC as the maximum acceptable value of SEM (11). Additional efforts in other quality assurance activities will be required to meet these expectations. Lack of generally agreed criteria on what constitutes ‘quality’ in work disability assessment hampers this challenging task further (38).

The interest in improving current practice becomes apparent from the international attention that was reached by a recent systematic review on the reproducibility of work disability evaluations with more than 20,000 full text hits (2). Until today, suitable instruments developed and validated in the setting of work disability assessment are missing which hinders the development of evidence-based policies (39). If instruments were available, the latitude of judgements would require explanation, training, calibration, and regular refreshers to maintain acceptable levels of reproducibility. Quality assurance will require more sophisticated, but easy-to-use monitoring and surveillance activities. Internet-based tools could be a promising approach.

Likewise, we need more studies comparing assessment strategy A versus B, including their precision on prognostic predictions like successful return-to-work. Initiatives are emerging (31, 32, 40–43), but they will require support from the insurance medicine community in order to succeed. Consorted efforts could help: researchers who provide methodological skills and experience in conducting studies, professional organisations who contribute content expertise, social insurers who advise about their knowledge needs and help with recruitment of claimants and funding, patient organisations who ensure the integration of the claimant perspective. Such consorted efforts would be able to generate the evidence required for improving practice.

In conclusion, content analysis revealed that RELY 1 did not succeed in integrating semi-structured functional interviewing as an integrated part of independent medical evaluations. The positive association between interviews with higher functional coverage and expert agreement on the claimants’ remaining WC is promising. It requires confirmation in prospective studies.

## Data Availability Statement

Raw data generated for this study are transcripts of patient interviews and they can not be made publicly available, due to patient confidentiality and privacy. However, the frequency tables of the coded interviews supporting the conclusions of this manuscript will be made available by the authors, without undue reservation, to any qualified researcher.

## Ethics Statement

The studies involving human participants were reviewed and approved by: All study procedures were approved by the cantonal ethics committees in Basel, Bern, Luzern, Zürich; the data protection officers of Basel-Stadt; Swiss National Science Foundation, Federal Social Insurance Office, Swiss National Accident Insurance Fund (Suva), and Disability Insurance Office in Zürich. All patients provided written informed consent according to procedures approved by the ethics committees. The patients/participants provided their written informed consent to participate in this study.

## Author Contributions

DA, WB, and RK conceived and designed the study. RD performed the transcription. DA, WB, RD, SK, and NV developed the category system and performed the coding. DA performed the analysis. All interpreted the findings. DA and RK drafted the manuscript. All authors contributed to the article and approved the submitted version. RK and DA are accountable for all aspects of the work.

## Funding

This secondary research draws on data of the RELY-studies (17, 19) and was funded by in-house funding. The RELY studies had been supported by grants from the Swiss National Science Foundation (project number 325130_144200), the Federal Social Insurance Office, and the Swiss National Accident Insurance Fund. The funders had no role in the design, data collection, analysis, or interpretation of the data.

## Conflict of Interest

After data collection had been finished (07/2016), RK became head of the Medical Competence Centre of Suva, Lucerne.

The authors declare that the research was conducted in the absence of any commercial or financial relationships that could be construed as a potential conflict of interest.

## Footnotes

1 Inter-rater reliability is the degree to which two or more raters are able to differentiate among subjects or objects under similar assessment conditions.

2 Inter-rater agreement is the degree to which two or more raters achieve identical results under similar assessment conditions.

3 Website to the RELY studies www.unispital-basel.ch/ebim/RELY

## Abbreviations

CI, confidence interval; ICC, Intraclass Correlation Coefficient; ICD 10, International Classification of Diseases (10th revision); IFAP, Instrument for Functional Assessment in Psychiatry; IQR, Interquartile range; MEDAS, Centre for Medical Assessments (Medizinische Abklärungsstelle); RELY, Reliable disability evaluation in psychiatry (acronym); SEM, Standard Error of Measurement; Suva, Swiss National Accident Insurance Fund; WC, Work capacity.

## References

[1] Anner J Kunz R Boer WD Reporting about disability evaluation in European countries. Disabil Rehabil (2014) 36(10):848–54. 10.3109/09638288.2013.821180 23919642

[2] Barth J de Boer W Busse JW Hoving JL Kedzia S Couban R Inter-rater agreement in evaluation of disability: systematic review of reproducibility studies. BMJ (2017) 356:j14. 10.1136/bmj.j14 28122727 PMC5283380

[3] Escorpizo R Stucki G Disability evaluation, social security, and the international classification of functioning, disability and health: the time is now. J Occup Environ Med (2013) 55(6):644–51. 10.1097/JOM.0b013e318297ae47 23722944

[4] Anner J Schwegler U Kunz R Trezzini B de Boer W Evaluation of work disability and the international classification of functioning, disability and health: what to expect and what not. BMC Public Health (2012) 12:470. 10.1186/1471-2458-12-470 22720978 PMC3432619

[5] Colomb E Dittmann V Ebner G Hermelink U Hoffmann-Richter U Kopp HG Qualitätsleitlinien für psychiatrische Gutachten in der Eidgenössischen Invalidenversicherung. [Guideline of the Swiss Society of Psychiatry and Psychotherapy]. Bern, Switzerland (2012).

[6] Baumberg Geiger B Garthwaite K Warren J Bambra C Assessing work disability for social security benefits: international models for the direct assessment of work capacity. Disabil Rehabil (2018) 40(24):2962–70. 10.1080/09638288.2017.1366556 28841811

[7] Riemer-Kafka G Independent Medical Evaluations for Insurance Medicine. Interdiciplinary medico-legal guidance. (Versicherungsmedizinische Gutachten) Chapter D: Potential role of the ICF in the evaluation of functional work capacity. 3rd ed Bern: Stämpfli (2017).

[8] Jeger J New Guidelines for Medical Assessments [Neue Leitlinien zur medizinischen Begutachtung]. Swiss Med J (Schweiz Aerztezeitung SaeZ) (2017) 98(17):525–6. 10.4414/saez.2017.05534

[9] Ebner G Colomb E Mager R Marelli R Rota F Guidelines for Independent Medical Evaluations of Mental and Psychosomatic Disorders in Insurance Medicine [Leitlinien für die Begutachtung psychiatrischer und psychosomatischer Störungen in der Versicherungsmedizin]. 3rd ed. (2016). www.psychiatrie.ch (last accessed 07.12.2019).

[10] Schandelmaier S Fischer K Mager R Hoffmann-Richter U Leibold A Bachmann MS Evaluation of work capacity in Switzerland: a survey among psychiatrists about practice and problems. Swiss Med Wkly (2013) 143:w13890. 10.4414/smw.2013.13890 24338835

[11] Schandelmaier S Leibold A Fischer K Mager R Hoffmann-Richter U Bachmann MS Attitudes towards evaluation of psychiatric disability claims: a survey of Swiss stakeholders. Swiss Med Wkly (2015) 145:w14160. 10.4414/smw.2015.14160 26295715

[12] Swiss Society of Rheumatology (2016). Guidelines for independent medical evaluations in rheumatology [Leitlinien für die rheumatologische Begutachtung] Retrieved from www.rheuma-net.ch.

[13] Brage S Donceel P Falez F EUMASS-Working-Group. Development of ICF core set for disability evaluation in social security. Disabil Rehabil (2008) 30(18):1392–6. 10.1080/09638280701642950 18850352

[14] Wiegand NM Belting J Fekete C Gutenbrunner C Reinhardt JD All Talk, No Action?: The Global Diffusion and Clinical Implementation of the International Classification of Functioning, Disability, and Health. Am J Phys Med Rehabil (2012) 91(7):550. 10.1097/PHM.0b013e31825597e5 22561387

[15] de Boer W Marelli R Hoffmann-Richter U Mager R Functional Assessment in Psychiatry. A Manual (Funktionsorientierte Begutachtung in der Psychiatrie: Eine Anleitung). Basel, Switzerland (2013).

[16] de Boer WEL Wijers JHL Spanjer J Beijl IVD Zuidam W Venema A Models for interviewing in insurance medicine [Gespreksmodellen in de verzekeringsgeneeskunde]. TVBV (2006) 14(1):19–26. 10.1007/BF03074300

[17] Kunz R von Allmen DY Marelli R Hoffmann-Richter U Jeger J Mager R The reproducibility of psychiatric evaluations of work disability: two reliability and agreement studies. BMC Psychiatry (2019) 19(1):205. 10.1186/s12888-019-2171-y 31266488 PMC6607597

[18] van Rijssen HJ Schellart AJM Anema JR de Boer WEL van der Beek AJ Systematic development of a communication skills training course for physicians performing work disability assessments: from evidence to practice. BMC Med Educ (2011) 11:28. 10.1186/1472-6920-11-28 21639871 PMC3138427

[19] Bachmann M de Boer W Schandelmaier S Leibold A Marelli R Jeger J Use of a structured functional evaluation process for independent medical evaluations of claimants presenting with disabling mental illness: rationale and design for a multi-center reliability study. BMC Psychiatry (2016) 16(1):271. 10.1186/s12888-016-0967-6 27474008 PMC4966817

[20] Kottner J Audige L Brorson S Donner A Gajewski BJ Hrobjartsson A Guidelines for Reporting Reliability and Agreement Studies (GRRAS) were proposed. J Clin Epidemiol (2011) 64(1):96–106. 10.1016/j.jclinepi.2010.03.002 21130355

[21] de Vet HC Terwee CB Dl K Bouter LM When to use agreement versus reliability measures. J Clin Epidemiol (2006) 59:1033–9. 10.1016/j.jclinepi.2005.10.015 16980142

[22] Dresing T Pehl T Praxisbuch Interview, Transkription & Analyse. Anleitungen und Regelsysteme für qualitativ Forschende. Marburg, Germany (2013).

[23] Strijbos J-W Martens RL Prins FJ Jochems WMG Content analysis: What are they talking about? Comput Educ (2006) 46(1):29–48. 10.1016/j.compedu.2005.04.002

[24] Hayes AF Krippendorff K Answering the Call for a Standard Reliability Measure for Coding Data. Commun Methods Meas (2007) 1(1):77–89. 10.1080/19312450709336664

[25] Krippendorff K Systematic and Random Disagreement and the Reliability of Nominal Data. Commun Methods Meas (2008) 2(4):323–38. 10.1080/19312450802467134

[26] Krippendorff KH Content Analysis: An Introduction to Its Methodology. Revised. ed SAGE (2012). 2012/29/Mai 456 p.

[27] Cohen J A power primer. Psychol Bull (1992) 112:155–9. 10.1037/0033-2909.112.1.155 19565683

[28] Spanjer J Krol B Brouwer S Popping R Groothoff JW van der Klink JJL Reliability and Validity of the Disability Assessment Structured Interview (DASI): A Tool for Assessing Functional Limitations in Claimants. J Occup Rehabil (2010) 20(1):33–40. 10.1007/s10926-009-9203-2 19779804 PMC2832901

[29] Spanjer J van der Mei S Cornelius B Brouwer S van der Klink J Effects of a training in the Disability Assessment Structured Interview on the interviews of Dutch insurance physicians. Disabil Rehabil (2016) 38(16):1632–41. 10.3109/09638288.2015.1106600 26679055

[30] Krohne K Brage S New rules meet established sickness certification practice: A focus-group study on the introduction of functional assessments in Norwegian primary care. Scand J Prim Health Care (2007) 25(3):172–7. 10.1080/02813430701267421 PMC3379777 17846936

[31] Bertilsson M Maeland S Love J Ahlborg G Jr. Werner EL Hensing G The capacity to work puzzle: a qualitative study of physicians’ assessments for patients with common mental disorders. BMC Fam Pract (2018) 19(1):133. 10.1186/s12875-018-0815-5 30060734 PMC6066915

[32] Østerås N Gulbrandsen P Benth J Hofoss D Brage S Implementing structured functional assessments in general practice for persons with long-term sick leave: a cluster randomised controlled trial. BMC Family Pract (2009) 10:31. 10.1186/1471-2296-10-31 PMC2688495 19419575

[33] Spanjer J Krol B Popping R Groothoff J Brouwer S Disability assessment interview: The role of detailed information on functioning in addition to medical history-taking. J Rehabil Med (2009) 41(4):267–72. 10.2340/16501977-0323 19247547

[34] de Boer WE Wind H van Dijk FJ Willems HH Interviews for the assessment of long-term incapacity for work: a study on adherence to protocols and principles. BMC Public Health (2009) 9:169. 10.1186/1471-2458-9-169 19490614 PMC2698854

[35] Gross DP Asante AK Miciak M Battié MC Carroll LJ Sun A A Cluster Randomized Clinical Trial Comparing Functional Capacity Evaluation and Functional Interviewing as Components of Occupational Rehabilitation Programs. J Occup Rehabil (2014) 24(4):617–30. 10.1007/s10926-013-9491-4 24374369

[36] Gross DP Asante AK Miciak M Battié MC Carroll LJ Sun A Are Performance-Based Functional Assessments Superior to Semistructured Interviews for Enhancing Return-to-Work Outcomes? Arch Phys Med Rehabil (2014) 95(5):807–15.e1. 10.1016/j.apmr.2014.01.017 24502839

[37] Marfeo EE Haley SM Jette AM Eisen SV Ni P Bogusz K Conceptual Foundation for Measures of Physical Function and Behavioral Health Function for Social Security Work Disability Evaluation. Arch Phys Med Rehabil (2013) 94(9):1645–52.e2. 10.1016/j.apmr.2013.03.015 23548543 PMC4010070

[38] de Boer WEL Quality of evaluation of work disability. Academic Thesis. Leiden, the Netherlands (2010).

[39] Werner EL Merkus SL Maeland S Jourdain M Schaafsma F Canevet JP Physicians’ assessments of work capacity in patients with severe subjective health complaints: a cross-sectional study on differences between five European countries. BMJ Open (2016) 6(7):e011316. 10.1136/bmjopen-2016-011316 PMC4947783 27417198

[40] Marfeo EE Ni P McDonough C Peterik K Marino M Meterko M Improving Assessment of Work Related Mental Health Function Using the Work Disability Functional Assessment Battery (WD-FAB). J Occup Rehabil (2018) 28(1):190–9. 10.1007/s10926-017-9710-5 PMC8935348 28477069

[41] Schleifer R Gamma A Warnke I Jabat M Rossler W Liebrenz M Online Survey of Medical and Psychological Professionals on Structured Instruments for the Assessment of Work Ability in Psychiatric Patients. Front Psychiatry (2018) 9:453. 10.3389/fpsyt.2018.00453 30319460 PMC6167551

[42] Tanner J Zeffiro T Wyss D Perron N Rufer M Mueller-Pfeiffer C Psychiatric Symptom Profiles Predict Functional Impairment. Front Psychiatry (2019) 10:37. 10.3389/fpsyt.2019.00037 30853916 PMC6396718

[43] Husabo E Monstad K Holmas TH Oyeflaten I Werner EL Maeland S Protocol for the effect evaluation of independent medical evaluation after six months sick leave: a randomized controlled trial of independent medical evaluation versus treatment as usual in Norway. BMC Public Health (2017) 17(1):573. 10.1186/s12889-017-4469-3 28615017 PMC5471703

