# Supplementary material for: Functional Interviewing Was Associated With Improved Agreement Among Expert Psychiatrists in Estimating Claimant Work Capacity: A Secondary Data Analysis of Real-Life Work Disability Evaluations
Source: Front Psychiatry. 2020 Jul 3;11:621. doi: 10.3389/fpsyt.2020.00621 (PMC7350701; doi:10.3389/fpsyt.2020.00621)
Supplement: Supplementary file 1 [file DataSheet_1.docx]

# Supplementary Material

Functional Interviewing Was Associated With Improved Agreement Among Expert Psychiatrists in Estimating Claimant Work Capacity: A Secondary Data Analysis of Real-Life Work Disability Evaluations

David Y. von Allmen, Sarah Kedzia, Raphael Dettwiler, Nicole Vogel, Regina Kunz* and Wout E. L. de Boer

*Correspondence: Regina Kunz [regina.kunz@usb.ch](mailto:regina.kunz@usb.ch)

Supplementary Table 1: Category System for medical/ general issues

Supplementary Table 2: Impact of low coverage versus high coverage interviews on work capacity, reliability and agreement, for last job and alternative work

Supplementary Table 3: Characteristics of psychiatrists and patients

Supplementary Table 4: Exemplary outtake from a functional interview in RELY 1

Supplementary Figure 1 - Figure: Relationship between coverage of functional topics and depth of enquiry during functional interviewing

**Supplementary Table 1: Category system for medical/general issues**

The category system for functional interviewing was extended by 15 general/medical issues plus two residual categories commonly addressed in disability assessments.

| **Categories for medical / general issues** | **General issues** | **Descriptions and examples** |
| --- | --- | --- |
| General medical complaints and symptoms | Specific complaints not explicitly related to work | Symptoms (type, localisation, severity) not reported in the context of work (e.g., excessive fatigue) |
|  | Exacerbating and attenuating circumstances | Circumstances that improve/worsen a symptom |
|  | Emotional-cognitive coping | Reaction to the experience of symptoms (e.g., experience of uselessness) |
|  | Residual category (general medical complaints) | Information associated with general medical complaints that cannot be assigned to the preceding three issues. |
| Cause/  intervention/ rehabilitation | Cause | Causes for existing health complaints (e.g., accident). |
|  | Intervention | Medical interventions against existing health complaints (e.g., physical therapy, medication, sick leave). |
|  | Rehabilitation | Participation in vocational reintegration. |
| Additional issues | Motivation to work | Commitment for reintegration |
|  | Health-related behaviour | Sports, diet, etc. |
|  | Life events | Marriage, child birth, divorce, death of family member or close friend, heavy injury, war, etc. |
|  | Future thinking | Wishes, hopes, goals etc. |
|  | Daily routine | Waking up, shower, breakfast, newspaper etc. |
|  | Personal development | Childhood development, change of personality, etc. |
|  | Residual category (additional issues) | Information not associated with the preceding six issues |
| Summary | General medical complaints | … |
|  | Cause/intervention/ rehabilitation | … |
|  | Additional issues | … |

**Supplementary Table 2: Impact of low coverage versus high coverage interviews reported for last job and alternative work in analogy to RELY 1,** on work capacity (WC), inter-rater reliability (intraclass correlation coefficient, ICC) and inter-rater agreement (standard error of measurement, SEM), (1)

|  | | Low coverage interviews  (95% CI) | High coverage interviews  (95% CI) | **Difference**  (Low coverage - high coverage interviews)  (95% CI) |
| --- | --- | --- | --- | --- |
| **Work capacity estimates,** expressed in % WC | Last job | 35.9% WC  (21.7 to 50.3) | 50.2% WC  (37.8 to 63.5) | -14.3% WC  (-4.5 to -27.6) |
|  | Alternative work | 46.1% WC  (31.0 to 61.2) | 63.0% WC  (52.6 to 73.4) | -16.9% WC  (-6.1 to -28.9) |
|  | |  | | |
| **Agreement as** SEM, expressed in % WC | Last job | 28.4% WC  (21.8 to 35.2) | 23.3% WC  (18.2 to 28.5) | 5.1% WC  (-3.3 to 13.8) |
|  | Alternative work | 28.1% WC  (22.1 to 34.2) | 20.6% WC  (16.3 to 25.0) | 7.5% WC  (0.2 to 15.1) |
|  | |  | | |
| **Reliability** as ICC expressed as ratio from 0 to 1 | Last job | 0.31  (0.06 to 0.55) | 0.41  (0.12 to 0.64) | -0.10  (-0.45 to 0.27) |
|  | Alternative work | 0.40  (0.10 to 0.63) | 0.38  (0.07 to 0.62) | 0.02  (-0.35 to 0.41) |

1. Kunz R, von Allmen DY, Marelli R, Hoffmann-Richter U, Jeger J, Mager R, et al. The reproducibility of psychiatric evaluations of work disability: two reliability and agreement studies. BMC Psychiatry. 2019;19(1):205.

**Supplementary Table 3: Characteristics of expert psychiatrists and claimants.**

Characteristics of psychiatrists and patients, including the main diagnoses of the claimants’ mental disorder(s) with impact on work capacity. Six claimants had been assigned two main diagnoses. Percentages are rounded to the nearest whole number.

| **Expert psychiatrists (N=12)** |  |
| --- | --- |
| Age: 31-40 / 41-50 / 51-60 / >60 years | 8% / 42% / 17% / 33% |
| Gender: male | 83% |
| Disability assessments in previous year: 5-20/ 21-50/ >50 | 17%/ 25%/ 58% |
| Professional experience |  |
| Years since board certification as psychiatrist, mean (SD) | 16.9 (11.1) |
| Years performing disability evaluations, mean (SD) | 13.3 (9.6) |
|  |  |
| **Claimants (N=30)** |  |
| Age in years, mean (SD) | 47.2 (8.6) |
| Gender: male | 57% |
| Nationality: Swiss/ other/ missing | 63%/ 23%/ 14% |
| Severity of mental disorder on a scale from 0-10, mean (SD), higher scores indicating more severe disorders | 5.3 (2.1) |
| Typicality of study claimant compared to other claimants seen by the expert: frequent/ semi-frequent/ rare | 36%/ 44%/ 20% |
|  |  |
| **Main diagnoses (ICD 10 classification)** | Number of diagnoses  N=36 |
| Organic (F0) | 11% |
| Psychoactive substance use (F1) | 3% |
| Mood disorders (F3) | 26% |
| Neurotic, stress-related and somatoform disorders (F4) | 19% |
| *thereof somatoform disorders (F45)* | 6% |
| Disorders of adult personality and behaviour (F6) | 11% |
| Claimants without diagnosis | 19% |

## Supplementary Table 4: Exemplary outtake from a RELY 1-assessment during functional interviewing. The expert enquired the patient’s health complaints related to work and her self-perceived work limitations.

| **Speaker** | **Content** |
| --- | --- |
| Interviewer | I would like to get to your last job now as a first step. And to the work as it was before the sick leave. It's been a while. Try to think your way back into this situation. And just tell me what it was like last year before the sick leave. What were your tasks? What it looked like exactly. |
| Patient | I was employed as a temporary employee and was responsible for opening master data. That means new article numbers with the new products. Then/ |
| Interviewer | Excuse me. I'm interrupting you now. What did the company do? What kind of company was that? |
| Patient | That was the company (…) in (…) and they manufactured (…) and (…) . |
| Interviewer | And in your responsibility was to open master data you have said. Can you tell me exactly what that is? |
| Patient | These are article numbers assigned to a product. And these are opened in the system. |
|  | |
| Interviewer | If you imagine now going back to work today. What would that look like? If you would go there today and you would have to resume your job now? |
| Patient | So actually if I think about it, I would now start the computer, as I did every day. I would have to look at these 50 mails that I got every day about/ sort them, set priority, it would not be possible for me at all. Maybe I would have to get up after the 10th mail and just run away, which is what I do today and at home. If it just gets too much for me, then I have to run away, switch off and do nothing. |
| Interviewer | This means that after about 10 mails you would then be/ |
| Patient | But not insanely complicated mails, but which I have to study and read a lot. |
| Interviewer | Would you stop because you get tired or is it the inner restlessness or tension? What is it then? What is it like? |
| Patient | It is actually the inner restlessness that I have. The nervousness. I have no patience anymore. Everything I had before is gone. |
| Interviewer | And that would then increase after 10 mails you would have processed? |
| Patient | Yes. |
|  |  |
| Interviewer | So if I may sum this up now: If you had to work now, you would have several difficulties. You couldn't concentrate on the e-mails. It would be hard for you to extract the essentials. Precise wordings. And you would have to stop or not finish some of the tasks because you get tired. You would develop such a strange feeling that you would feel different. |
| Patient | Especially what happens after a great effort, when I get into a kind of resting phase, which is just almost thoughtless, I have like little strange seizures. The chest starts to hurt. And then it goes towards the neck. Like someone squeezing the air out of me. And then it radiates into the whole arm. And then/ |
| Interviewer | Pain? |
| Patient | Pain. That I also lose the feeling. And then the arm just falls down. And I have no more strength. That's one thing. And the other is losing body control. That it pulls me to the left while standing. That I no longer have the strength. I have to start supporting myself somewhere or fall in the worst case. And that happened to me for the first time during rehab. And since then the situation has changed completely. |

## Supplementary Figure 1: Relationship between coverage of functional key topics and depth of enquiry during functional interviewing. Linear regression analysis revealed a positive relationship of R=0.76 (*p*<0.001) between the number of functional key topics covered at least once by either psychiatrist or patient during the interview and the sum of functional coding units assigned to key topics. Example: Interview A had 83 functional coding units assigned to address 11 of 38 possible key topics (i.e., 7.5 functional coding units per key topic). Interview B had 537 functional coding units assigned to address 29 of 38 possible key topics (i.e., 18.5 functional coding units per key topic). Accordingly, with an increasing number of key topics covered in the interview, the sum of functional coding units assigned to those topics increased above-average indicating a more in-depth conversation about the patient’s functional capacity.


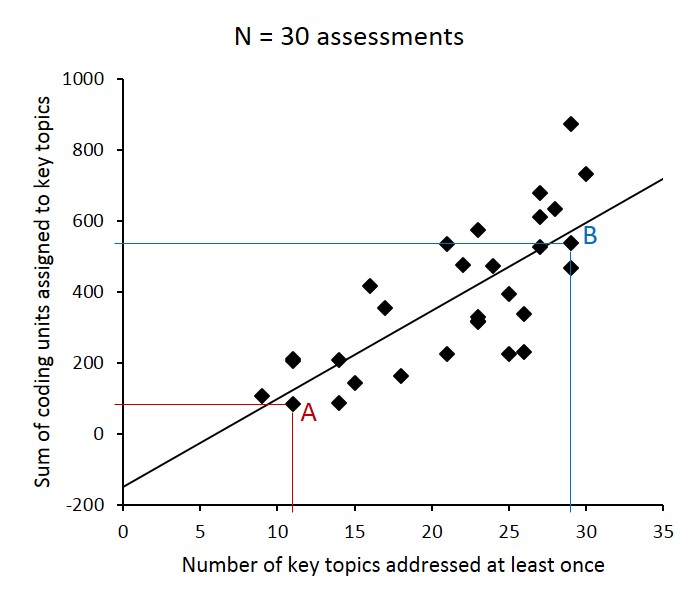


(R = 0.76, *p* < 0.001

## 
